# One-stage posterior C2 and C3 pedicle screw fixation or combined anterior C2-C3 fusion for the treatment of unstable hangman’s fracture

**DOI:** 10.3892/etm.2013.898

**Published:** 2013-01-16

**Authors:** JINGCHEN LIU, YE LI, YUNTAO WU

**Affiliations:** Department of Spine Surgery, The First Hospital of Jilin University, Changchun, Jilin 130021, P.R. China

**Keywords:** hangman’s fracture, C2, C3, pedicle screw, instability

## Abstract

The present study aimed to evaluate the effect of using one-stage posterior C2 and C3 pedicle screw fixation or combined anterior C2-C3 fusion in the treatment of unstable hangman’s fracture. A total of 13 patients with unstable hangman’s fractures underwent C2 and C3 pedicle screw fixation, lamina interbody fusion or combined anterior C2-C3 fusion and imaging examinations to evaluate the fracture fixation and healing condition at three days and three months following surgery. Postoperative X-ray and computed tomography (CT) results showed high fracture reduction, good internal fixation position and reliable fracture fixation. The three-month postoperative CT showed good vertebral fracture healing. C2 and C3 pedicle screw fixation has a good curative effect in the treatment of unstable hangman’s fracture. The direct fixation of the fracture enables early ambulation by the patients.

## Introduction

Hangman’s fracture, also called traumatic spondylolisthesis of the axis, is a common fracture of the second cervical vertebra. In 1985, Levine and Edwards (1) classified hangman’s fracture into four types.

Levine-Edwards type I is described as a stable fracture and is treated with conservative treatment. Levine-Edwards types II, IIA and III are mainly treated surgically using a number of methods. Unstable hangman’s fracture is an injury that usually occurs in the anterior longitudinal ligament, posterior longitudinal ligament, C2-C3 intercalated disc and atlantoaxial isthmus and is caused by hyperextension force (2) on the third spinal columns. Anterior or posterior stable fixation surgery is usually performed. Anterior surgery typically includes C2-C3 disc excision, interbody fusion, plate fixation (due to the simple surgery) and short-segment fusion (3). However, this procedure does not directly fix the separate isthmus of the atlantoaxial pedicle. Posterior surgery was previously performed using C1-C3 wire fixation but required postoperative Halo-vest fixation (4). The atlantoaxial isthmus fracture was treated with direct screw fixation by Bristol *et al*(5) to retain the atlanto-axial activity. However, the stability of the C2-C3 discs was not maintained by this approach due to disc damage (6,7). A number of studies on C2 isthmus screw fixation combined with C3 lateral mass screw fixation, as well as C1 pedicle combined with C3 lateral mass screw fixation, have been reported (8). However, the combination of C1 pedicle and C3 lateral mass screw fixation is not considered desirable since it results in a significant loss of C1-C2 activity. By contrast, the combination of C2 isthmus and C3 lateral mass screw fixation is a more effective fixation method. With the increasing use of the pedicle screws, combined C2-C3 pedicle screw fixation has improved the stability of C2-C3 in hangman’s fracture. Duggal *et al*(9) conducted a biomechanical study and observed that C2 isthmus screw fixation combined with C3 screw fixation was more stable than anterior plate fixation. Whether the surgery is anterior or posterior, the purpose is the fusion of the C2-C3 segments. A higher degree of immobilisation leads to higher fusion rates. Thus, a strong fixation may increase the fusion rate of hangman’s fracture. In clinical treatments, the application of posterior C2 and C3 fixation to a highly unstable hangman’s fracture results in the aggravation of the forward displacement of C2 due to the intraoperative prone position, particularly with the extremely unstable state of C2. This aggravation causes iatrogenic injury and may lead to extremely negative consequences. Therefore, in the current study, anterior C2-C3 interbody fusion was attempted first, followed by the procedure involving direct one-stage posterior C2 and C3 pedicle screw fixation in the fracture at the isthmus position.

The present study aimed to evaluate the feasibility of using C2 and C3 pedicle screw fixation or combined anterior C2-C3 interbody fusion for the treatment of unstable hangman’s fractures through clinical case research. The present study also evaluated the performance of magnetic resonance imaging (MRI) in determining the stability of hangman’s fractures. Whether the original classification system requires updating given the increasingly advanced examination methods currently available was also investigated.

## Materials and methods

### General data

A total of 13 patients with unstable hangman’s fracture were treated between March 2009 and March 2011. The group consisted of 10 males and three females aged 23–48 years old (average age, 39.4 years old). The cause of injury was traffic accidents for eight cases, fall accidents for three cases and two were bruises. All patients underwent preoperative X-ray, computed tomography (CT) and MRI examination. X-ray examination was used to determine the standard anteroposterior, lateral and opening positions. CT examination further revealed the dislocation, angulation and fracture conditions. MRI examination was used to evaluate the C2-C3 intervertebral disc injury conditions, as well as the presence of free intraspinal intervertebral discs and spinal cord compression. The fractures were classified as follows based on the Levine-Edwards classification: one case of type I with intervertebral disc damage, as determined by MRI which was thereafter classified as a potentially unstable hangman’s fracture; seven cases of Levine-Edwards type II with small angulation, in which C2 was clearly displaced forward compared with C3; four cases of Levine-Edwards type IIA with clear C2-C3 intervertebral angulation; and one case of Levine-Edwards type III, in which C2 was displaced forward by ∼5.3 mm and the right C2-C3 articular process suffered fracture and dislocation. All patients experienced neck pain with neck rotation, as well as limited flexion and extension. The preoperative score of the spinal cord function was grade E according to the ASIA classification. The patients all underwent preoperative skull traction using a traction weight that ranged from 2 to 5 kg. The end traction indices were C2-C3 shift <3 mm and angulation <5° (Table I). The present study was conducted in accordance with the Declaration of Helsinki and with approval from the Ethics Committee of the First Bethune Hospital of Jilin University. Written informed consent was obtained from all participants.

### Surgical method

The one case of Levine-Edwards type I received simple posterior C2 and C3 pedicle fixation and lamina interbody fusion. The other patients all underwent anterior C2-C3 intervertebral disc excision, decompression and reduction, as well as interbody implantation of an autologous iliac bone graft and internal fixation with a steel plate. Afterward, these patients underwent posterior C2 and C3 pedicle screw fixation and lamina interbody fusion.

The patients underwent anterior C2-C3 intervertebral disc excision, interbody fusion with autologous iliac bone grafts and anterior cervical plate fixation. The patients were in the supine position with their shoulders and necks supported, resulting in the slight hyperextension of their necks. The skull tractions were not removed and the axial tractions were retained at a weight of 2 kg. The anterior cervical transverse incision exposed the C2-C3 clearance, thus exposing the torn anterior longitudinal ligament, red surface, opened C2-C3 clearance and ruptured intervertebral disc of the majority of patients. The ruptured intervertebral disc was resected to expose the torn posterior longitudinal ligament. The ruptured intervertebral disc tissues in the spinal canal were cleared away. The autogenous iliac bone corrected to a suitable gap size was removed and placed inside the C2-C3 clearance. A steel plate of an appropriate size was fixed in front of the C2 and C3 centrum. A drainage tube was placed after flushing the incision and removed within 48 h after surgery depending on the drainage flow.

Following the anterior surgery, the skull traction was removed. The patients were in the prone position, with their heads fixed with Mayfield frames. The neck was in slight flexion with the posterior midline incision to expose the C2 and C3 bilateral vertebral plates fully. The C2 fracture was then exposed. A 2-mm abrasive drill was used to grind the bone cortex in the midpoint of the C2 lower articular process. Grinding was intraoperatively continued inwards and upwards through the fracture line to the anterior cortex of the vertebral body. After the abrasive drilling was stopped, a screw tap was used to expand the screw channel through the fracture line. A larger screw tap was used to expand the channel behind the fracture line further. The depth of the screw channel was measured and the pedicle screws with diameters of 3.5 mm were then screwed into the channel. The C3 spinous process was fixed against the torque force of the screw, whereas the pedicle screw crossed the fracture line to prevent structural dislocation in the fracture. After the C3 pedicle screw fixation, C2/3 pedicle screws were connected with a connecting rod, the sclerotin of the C2/3 spinous process was removed and the bone cortex of the C2/3 vertebral plate was removed via abrasive drilling. The removed spinous process was formed into bone fragment tablets, which were then paved on the C2/3 vertebral plate. The drainage tube was then placed into the closed incision and removed within 48 h after the surgery. One case was given a single posterior surgery (Table I).

The patients received postoperative antibiotics for three to five days. They were able to sit up or walk with the protection of a neck brace following the postoperative removal of the drainage tube. The neck brace was routinely used for two weeks. X-ray examination was performed three days and three months after surgery to assess the resetting conditions of C2 and C3. CT examination was performed three days and three months after surgery to evaluate the healing conditions of the fracture.

## Results

**Figure 1.**
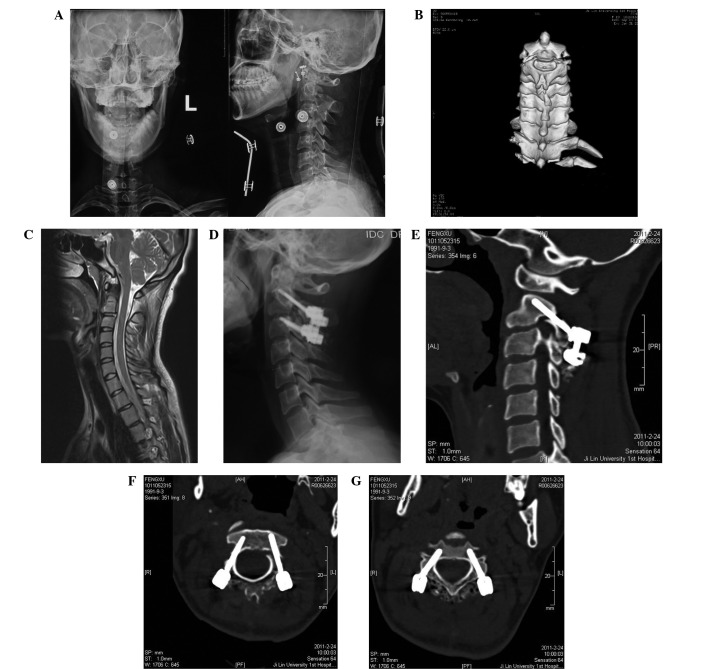
Hangman’s fracture type I. (A) X-ray film showing a type I fracture; (B) three-dimensional CT scan showing the fracture form; (C) MRI scan showing the C2-C3 disc damage at the back and anterior lower margin of the C2 fracture; (D) postoperative lateral X-ray film; (E) postoperative vertical CT; (F) transverse C2; and (G) transverse C3. CT, computed tomography; MRI, magnetic resonance image.

**Figure 2.**
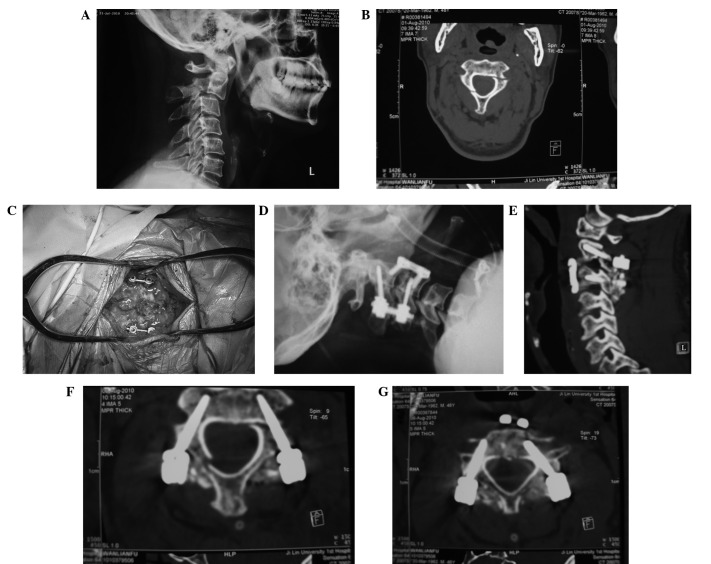
Hangman’s fracture type IIA. (A) X-ray film showing a type IIA fracture; (B) CT scan showing the fracture form; (C) intraoperative screw placement; (D) postoperative lateral X-ray film; (E) postoperative vertical CT; (F) transverse C2; and (G) transverse C3. CT, computed tomography.

The mean surgery time of the combined anterior and posterior surgery was ∼4.5 h. Haemorrhaging of ∼200 ml occurred, but no blood transfusion was performed. The simple posterior surgery lasted 90 min. No intraoperative or postoperative spinal cord or vertebral artery injury with well-healed incision was observed. Postoperative X-ray and CT scans showed that all fractures exhibited osseous healing without observable cervical functional limitations. A total of 52 screws were placed into 13 patients (each patient had 4 pedicle screws, 2 C2 pedicle screws and 2 C3 pedicle screws). Postoperative CT examination showed that one C2 pedicle screw was partially external (1/26), with a screw placement accuracy of 96%. Two C3 pedicle screws were partially external (2/26), with a screw placement accuracy of 92%. No internal fixation was loosened or fell off in the follow-up period. At the one-year follow-up, the bone graft was observed to be fused and the fracture line disappeared (Figs. 1 and 2). One patient continued to experience pain at the back of the neck at nine months following surgery. This pain may have been caused by scar contracture. The pain decreased following physical therapy.

## Discussion

At present, a number of controversies surround the treatment of hangman’s fracture. The early use of a large number of tractions and their subsequent reduction achieved satisfactory results in certain studies (10–12). However, Vaccaro *et al*(12) retrospectively analysed the early application of Halo fixation in the treatment of hangman’s fracture types II and IIA. A total of 27 type II cases and four type IIA cases were included in the study. Of these, 21 type II cases achieved a high fusion effect, whereas the remaining six type II cases and four type IIA cases exhibited failed fusion and required further surgery. The authors’ study showed that the angulations of the further surgery were ≥12°. Coric *et al* reported (13) that 60% of type II and type IIA fractures suffer from anterior C2/3 instability and cause persistent pains in the neck in the absence of strong fixation. Halo fixation generally requires > three months to complete; this long treatment time is a disadvantage.

Anterior surgery involves C2-C3 intervertebral disc excision, autologous iliac bone implantation or implantation of other materials within the fracture space followed by fixation with steel plates (5,14). The advantage of this surgery is the excision of the cataclastic intercalated disc, particularly the removal of the disc tissue in the spinal canal. However, simple anterior surgery does not fix the fracture position and often requires longer postoperative external fixation (14).

Posterior surgery consists of a number of procedures, including C2 cervical pedicle screw fixation, combined C2 pedicle and C3 lateral screw fixation, posterior fixation extended to C1 and even occipital-cervical fusion (2,15–18). Simple C2 pedicle screw fixation in posterior surgery does not remove the C2-C3 intervertebral instability. Therefore, simple C2 pedicle screw fixation in the treatment of unstable hangman’s fracture is not recommended. In addition, simple posterior surgery does not fix the C2-C3 cataclastic intercalated disc in the spinal canal or correct the advanced secondary spinal stenosis. The major concern is the unstable hangman’s fracture that usually accompanies C2 forward displacements. If single posterior surgery is performed in the prone position, this may further aggravate the dislocation and damage to the spinal cord, which may have negative consequences. Moreover, the forward thrust of the C2 screw placement may increase the dislocation.

In the present study, patients with unstable hangman’s fracture underwent one-stage anterior C2-C3 intervertebral disc excision, interbody fusion with autologous bone grafts, internal plate fixation and posterior C2 and C3 pedicle screw fixation. Twelve patients regained immediate postoperative stability and were able to stand and walk early, increasing their confidence. The imaging results showed high fracture reduction and fusion. Li *et al*(19) systematically analysed treatment methods for hangman’s fracture and observed that single anterior and single posterior surgeries have advantages and disadvantages. Duggal *et al*(9) and Kim *et al*(20) demonstrated in a biomechanical study that posterior fixation is more stable than anterior fixation. However, the majority of surgeons are more willing to choose anterior surgery due to the high risk and difficult surgery of posterior fixation (4,13,14). Therefore, the present study aimed to determine whether the simultaneous application of one-stage anterior and posterior fixation may be used to achieve three-column fixation and result in higher physiological stability. With the lower cost of internal fixation, patients are no longer concerned with the medical expense and instead consider which treatment has a greater curative effect. Reports concerning one-stage anterior-posterior surgery remain scarce. The present study used strong one-stage anterior and posterior fixation to achieve good effects.

In this procedure, the placement of the C2 pedicle screw is difficult due to the presence of a bilateral fracture with significant activity in the structures behind the fracture lines. The placement must be performed without temporary fixed installation, particularly when placing the first C2 pedicle. Although all cases used ordinary, fully-threaded cervical pedicle screws with diameters of 3.5 mm, the threads used behind the fracture line to increase the aperture had screw diameters of 4.0 mm. The threads used in the vertebral body to fix the rear C3 spinous process in front of the fracture line were suitable screws with diameters of 3.5 mm. Screws with diameters of 3.5 mm were then tightened to exert a pressure effect. The two steps were skipped when tension screws were used.

Levine and Edwards classified hangman’s fractures into four types. Types II, IIA and III are all generally considered to be unstable fractures. However, we suggest that the aforementioned classification system is based on a previous X-ray classification. Advancements in inspection equipment have led to the development of MRI, which is able to detect signal changes in the intervertebral discs with high sensitivity. X-ray analysis is unable to detect the shifts or angulation in a number of type I patients. However, MRI detected a disc rupture, which was considered as a potentially unstable fracture. The following are the characteristics of unstable hangman’s fracture: i) occurrence of a cervical C2 dislocation or cervical C2 and C3 disc destruction accompanied by cervical C2 isthmus or vertebral plate and accessory fractures; ii) the bulbar end is in the spinal canal. The majority of patients succumb at the time of injury due to respiratory and central circulation damage. Patients taken to the hospital usually lack system symptoms in addition to cervicodynia; and iii) conservative treatment is invalid and dangerous due to the absence of blood supply from the disc, which is unable to repair itself. Therefore, all hangman’s fracture patients should undergo cervical MRI examination to evaluate the C2-C3 disc damage and to determine whether the cataclastic intercalated disc protrudes into the spinal canal. MRI examination is particularly crucial for determining whether the intercalated disc is damaged in Levine-Edwards type I patients.

The present study included one Levine-Edwards type I patient with intervertebral disc injury, which was considered as a potentially unstable fracture. The patient was a young woman whose disc retained a certain degree of stability. The patient and her family were informed of the conservative and surgical treatments and they selected single posterior fixation to treat the patient’s condition. The patient and her family were satisfied with the postoperative effect. Related reports on this type of fracture are unavailable in the literature. In addition, MRI examination has rarely been used in the preoperative diagnosis of hangman’s fracture in previous studies. Therefore, further studies must be conducted concerning the application of MRI in the classification of hangman’s fracture as well as in the determination of fracture stability. In the present study, the merits of treatment and clinical examination of potentially unstable fractures were discussed.

Certain Levine-Edwards I cases show intervertebral disc injuries that exhibit potential instability. Further study must be conducted to determine whether these patients require surgery.

The application of combined anterior-posterior surgery for Levine-Edwards types II, IIA and III results in strong fixation and immediate stability, which allow patients to sit up or walk immediately after surgery and return to normal life as soon as possible. The bone graft is fixed to obtain a high fusion rate.

## Figures and Tables

**Table I.** Data of 13 patients.

Patient number	Type (gender)	Angulation (°)	Displacement (mm)	MRI diagnosis	Surgical strategy
1	I (F)	5	0.0	C2-C3 disc disruption	C2 and C3 pedicle screw fixation
2	II (M)	20	4.3	C2-C3 disc disruption	Anterior fusion + C2 and C3 pedicle screw fixation
3	II (F)	16	3.5	C2-C3 disc disruption	Anterior fusion + C2 and C3 pedicle screw fixation
4	II (M)	12	6.5	C2-C3 disc disruption	Anterior fusion + C2 and C3 pedicle screw fixation
5	II (M)	18	7.3	C2-C3 disc disruption	Anterior fusion + C2 and C3 pedicle screw fixation
6	II (F)	11	10.0	C2-C3 disc disruption	Anterior fusion + C2 and C3 pedicle screw fixation
7	II (M)	25	5.5	C2-C3 disc disruption	Anterior fusion + C2 and C3 pedicle screw fixation
8	II (M)	12	4.6	C2-C3 disc disruption	Anterior fusion + C2 and C3 pedicle screw fixation
9	IIA (M)	25	3.0	C2-C3 disc disruption	Anterior fusion + C2 and C3 pedicle screw fixation
10	IIA (M)	39	2.7	C2-C3 disc disruption	Anterior fusion + C2 and C3 pedicle screw fixation
11	IIA (M)	32	2.0	C2-C3 disc disruption	Anterior fusion + C2 and C3 pedicle screw fixation
12	IIA (M)	23	2.9	C2-C3 disc disruption	Anterior fusion + C2 and C3 pedicle screw fixation
13	III (M)	18	5.3	C2-C3 disc disruption	Anterior fusion + C2 and C3 pedicle screw fixation

Type, fracture type according to the Levine-Edwards classification; F, female; M, male; MRI, magnetic resonance imaging.
